# Dissociation in patients with non-affective psychosis: Prevalence, symptom associations, and maintenance factors

**DOI:** 10.1016/j.schres.2021.11.008

**Published:** 2022-01

**Authors:** Emma Černis, Andrew Molodynski, Anke Ehlers, Daniel Freeman

**Affiliations:** a University of Oxford Department of Psychiatry, Warneford Hospital, Oxford OX3 7JX, United Kingdom; b Oxford Health NHS Foundation Trust, Warneford Hospital, Oxford OX3 7JX, United Kingdom; c Oxford Centre for Anxiety Disorders and Trauma, Department of Experimental Psychology, University of Oxford, The Old Rectory, Paradise Square, Oxford OX1 1TW, United Kingdom

**Keywords:** Dissociation, Psychosis, Network analysis, Psychological mechanisms, Prevalence, Depersonalization

## Abstract

Dissociation is problematic in its own right for patients with psychosis but may also contribute to the occurrence of psychotic experiences. We therefore set out to estimate in a large cohort of patients with psychosis the prevalence of dissociative experiences, and assess using network models the relationships between dissociation, its potential maintenance mechanisms, and mental health symptoms.

902 patients with non-affective psychosis attending UK mental health services participated. Both an undirected model and a partially directed network model were estimated to identify potential relationships between ‘felt sense of anomaly’ dissociative experiences, paranoia, hallucinations, psychological wellbeing, sleep, and six potential maintenance mechanisms (affect intolerance, perseverative thinking, general self-efficacy, alexithymia, cognitive appraisals, and cognitive-behavioural responses to dissociation).

617 patients (65.4%) had experienced at least one dissociative symptom regularly over the past fortnight, with the average number experienced being 8.9 (SD = 8.0). Dissociation had direct relationships with paranoia, hallucinations, low psychological wellbeing, cognitive appraisals, cognitive-behavioural responses to dissociation, perseverative thinking, and low alexithymia. Dissociation was a probable cause of hallucinations (94.21% of 50,000 sampled directed acyclic graphs), with a trend towards also being a cause of paranoia (86.25% of 50,000 sampled directed acyclic graphs).

Approximately two-thirds of patients with psychosis experience regular dissociative experiences. Dissociation is associated with low psychological wellbeing, and it is likely to have a direct causal influence on psychotic symptoms. Catastrophic cognitive appraisals, cognitive-behavioural responses to dissociation, factors related to affect sensitivity, and perseverative thinking may contribute to the occurrence of dissociation.

## 1 Introduction

‘It was just the most- very, very lonely time […] because everything else is going on in my head, and this disconnected feeling.’

– Participant quotation (Černis et al., 2020b).

Dissociative experiences can cause significant distress, feelings of being overwhelmed, and worsening of psychotic experiences for patients with psychosis. (Černis et al., 2020b; Varese et al., 2011; Longden et al., 2020). Dissociation, across mental health presentations, is not well-understood by clinicians (Bailey and Brand, 2017). It is also a challenging area for research, with ongoing debate regarding classification and measurement likely to have hindered determination of the causes of dissociation. In the current study, therefore, we aimed to estimate in a large psychosis patient group the prevalence of a precisely-defined type of dissociation, and assess using network analyses the relationships between dissociation, its potential maintenance mechanisms, and mental health symptoms, including psychotic symptoms.

There have been three large clinical studies of dissociation in the context of psychosis. The largest reports longitudinal associations over decades between first-rank psychosis symptoms, depersonalisation, and derealisation in 167 schizophrenia spectrum and 156 ‘other psychosis’ patients (Humpston et al., 2020). The other two studies focus on the relationship between dissociation and trauma. Schäfer et al. (2012), in a study with 145 patients, found dissociative symptoms were predicted by positive psychotic symptoms at admission, and childhood sexual abuse. Schalinski et al. (2019), in a study with 180 patients, found dissociative symptoms mediated the relationship between childhood trauma and psychotic symptoms.

All three studies used different assessments of dissociation. Used interchangeably to signify either a symptom or an aetiological process, the term ‘dissociation’ has attracted multiple definitions incorporating a diverse range of experiences that have led to use of different assessment instruments (Černis et al., 2021). There is now increasing recognition that dissociation may comprise multiple distinct experiences (Holmes et al., 2005), and hence that progress will be hindered without greater specificity in conceptualisation and assessment. In our own work, we have used large general population datasets to identify a subgroup of dissociative experiences with the core phenomenology of a ‘felt sense of anomaly’ (FSA) (Černis et al., 2021). A subjective experience of strangeness may be felt in a range of ways, such as disconnection, automaticity, unreality, or unfamiliarity (Černis et al., 2020b). Furthermore, the experience may affect one or more of various domains, including the body, perception, identity, and cognition. This means that a broad range of experiences falls under the FSA subgroup, some of which may be described in terms of detachment/compartmentalisation (Holmes et al., 2005) or depersonalisation/derealisation (Sierra and Berrios, 1997, Sierra and Berrios, 1998).

We drew on our findings in the general population to test psychological processes that may lead to the occurrence and maintenance of dissociative experiences (Černis et al., submitted). Factors that may lead to dissociation include catastrophic cognitive appraisals, counter-productive safety-seeking responses, low general self-efficacy, alexithymia, and affect intolerance (Hunter et al., 2003; Černis et al., 2020b; Evren et al., 2012; Ó Laoide et al., 2018). Dissociation may be maintained by a process whereby changes in arousal are experienced as aversive due to heightened affect sensitivity and perceived low coping ability. Such experiences are therefore met with a negative cognitive-behavioural response, reinforced by counterproductive management strategies (such as avoidance) and heightened sensitivity. Additionally, previous literature (Barton et al., 2018; van Heugten–van der Kloet et al., 2015) has demonstrated associations between poor sleep and dissociation.

Therefore, the key aims of this study are to establish the prevalence of FSA-dissociative experiences in patients with psychosis, examine relationships with paranoia and hallucinations, and identify potential psychological causes of dissociation in order to inform therapeutic approaches. The aim of understanding dependencies between multiple variables at once – symptoms and their possible mechanisms – is one best met through network analysis. Such analyses are gaining increasing popularity in mental health research because they are well-suited to exploring complex sets of correlated variables (Robinaugh et al., 2019) and generating novel testable hypotheses regarding directions of causal effect. It should be noted, however, that there are significant challenges to identifying maintenance mechanisms using network analyses. Gaussian graphical models do not indicate direction of effect, and an assumption of the Directed Acyclic Graphs (DAGs) analysis is that relationships between variables are acyclic. As García-Velázquez et al. (2020) observe, this assumption constitutes an ‘oversimplification in the context of mental health’, since most relationships are likely to be reciprocal to some degree. Accordingly, it is anticipated that ambiguous directions of effect between proposed potential maintenance factors and dissociation are more likely than robust single directions of effect.

## 2 Methods

### 2.1 Design

The design was a cross-sectional self-report questionnaire study. Ethical approval was received from London – City and East NHS Research Ethics Committee (19/LO/1394).

### 2.2 Participants

Participants were recruited from thirty-six NHS trusts in England. Inclusion criteria were: age 16 years or over, currently under the care of an NHS mental health service, with a diagnosis of non-affective psychosis, and willing and able to give informed consent to participate. Exclusion criteria were: insufficient English language to complete the questionnaires with support, or an affective psychosis diagnosis.

Recruitment ran between 18th October 2019 and 19th March 2020. Datasets from 1038 participants were returned. Only cases with low levels of missing data in every measure (less than or equal to 20% missing) were retained for analysis, resulting in a participant group of 902 patients (Table 1).

**Table 1:** Demographic and clinical data for the participant group (*n* = 902).

Demographic and clinical information		n (%)
Gender	Female	269 (29.82%)
	Male	627 (69.51%)
	Other	4 (0.44%)
	Missing data	2 (0.22%)
Ethnicity	White (any)	619 (68.63%)
	Black (any)	147 (16.29%)
	Asian (any)	84 (9.31%)
	Mixed/multiple	33 (3.66%)
	Other	14 (1.55%)
	Missing data	5 (0.55%)
Diagnosis	Schizophrenia	579 (64.19%)
	Schizoaffective disorder	135 (14.97%)
	Delusional disorder	11 (1.22%)
	First episode psychosis	94 (10.42%)
	Psychotic disorder NOS	79 (8.76%)
Care team type	Inpatient	237 (26.27%)
	Outpatient	665 (73.73%)
	of which early intervention	110 (12.20%)

### 2.3 Measures

Cronbach's alphas for each scale are shown in Table 2. All scales demonstrated good or excellent internal consistency in this group.

**Table 2:** Means and standard deviations for each scale (n = 902).

Scale	Sample mean (SD)	Cronbach's alpha for this sample	Scale min – max score
Černis Felt Sense of Anomaly scale	40.56 (30.59)	0.97	0–140
Cognitive Appraisals of Dissociation	18.84 (13.19)	0.93	0–52
Responses to Dissociation	13.22 (4.80)	0.69	0–24
Green et al. Paranoid Thoughts Scale (Revised)*Persecution subscale*	13.94 (12.14)	0.94	0–40
Specific Psychotic Experiences Scale *Hallucinations subscale*	16.87 (16.00)	0.93	0–55
General Self-Efficacy scale	27.08 (7.36)	0.92	10–40
Perseverative Thinking Questionnaire	45.35 (16.31)	0.96	15–75
Affect Intolerance Scale	118.50 (34.89)	0.95	30–180
Online Alexithymia Questionnaire	31.96 (8.69)	0.79	11–55*
Warwick-Edinburgh Mental Wellbeing Scale	44.93 (12.75)	0.94	14–70
Sleep Condition Indicator	4.49 (2.93)	0.83	0–8

#### 2.3.1 Affect Intolerance Scale (AIS; Stapinski et al., 2014)

The Affect Intolerance Scale assesses respondents' attitudes towards negative emotions using 30 items rated on Likert scales from 1 “strongly disagree” to 6 “strongly agree”. Items form two factors: “threat expectancy” (“Once I have negative feelings, I worry that they will get worse”) and “avoid/suppress” (“I should avoid negative feelings”). Higher scores indicate greater intolerance of negative affect.

#### 2.3.2 Černis Felt Sense of Anomaly scale (ČEFSA; Černis et al., 2021)

The ČEFSA measures dissociative experiences with a core phenomenological experience of a felt sense of anomaly (FSA) using 35 items (“the world around me seems unreal”). Items are rated for the past two weeks on Likert scales from 0 “never” to 4 “always”. Higher scores indicate higher levels of FSA-dissociation.

#### 2.3.3 Cognitive Appraisals of Dissociation (CAD-P; Černis et al., 2020a)

The CAD-P measures cognitive appraisals of dissociative experiences. Respondents answer according to how they think when “feeling strange, disconnected, unreal or ‘dissociated’”. Thirteen items (“This might last forever”) are rated on Likert scales from 0 “never” to 4 “always”. Higher scores indicate more frequent occurrence of catastrophic appraisals in response to dissociative experiences.

#### 2.3.4 General Self-Efficacy Scale (GSE; Schwarzer and Jerusalem, 1995)

The General Self-Efficacy scale comprises ten items (“I can usually handle whatever comes my way”) rated for ‘how true … they are of you in general’ on four-point Likert scales from 1 “not at all true” to 4 “exactly true”. Higher scores indicate greater self-efficacy.

#### 2.3.5 Online Alexithymia Questionnaire (OAQ-G2 adapted; Thompson, 2007)

An adapted version of the OAQ was used to measure alexithymia. This comprised eleven items forming three factors: “difficulty identifying feelings” (“When asked which emotion I'm feeling, I frequently don't know the answer”); “difficulty describing feelings” (“I can describe my emotions with ease”); and “externally-oriented thinking” (“I prefer doing physical activities with friends rather than discussing each others' emotional experiences”). Items are rated from 1 “strongly agree” to 5 “strongly disagree” (with one reverse-coded item). Higher scores indicate greater difficulty identifying, naming, and acknowledging one's emotional state.

#### 2.3.6 Perseverative Thinking Questionnaire (PTQ; Ehring et al., 2011)

The PTQ is a 15-item measure of repetitive negative thinking. Items ask how the respondent ‘typically’ thinks about negative experiences or problems, rated from 0 “Never” to 4 “almost always”. Higher scores indicate higher levels of ruminative thinking.

#### 2.3.7 Responses to Dissociation (RTD; Černis et al., submitted)

Cognitive-behavioural responses to dissociation were measured using six items (“I try to keep busy”). Higher scores on the RTD indicate greater use of such responses to dissociation. Items are rated from 0 “never” to 4 “always” for how the respondent typically acts when feeling dissociated.

#### 2.3.8 Revised Green et al. Paranoid Thoughts Scale (R-GPTS Persecution; Freeman et al., 2019)

The R-GPTS assesses paranoia using two subscales: ideas of reference and persecution. The persecution subscale of the R-GPTS was used in this study. This comprises ten items (“Certain individuals have had it in for me”), rated over the past month on five-point Likert scales from “0 - not at all” to “4 – totally”. Higher scores indicate higher levels of paranoia: a score above 18 indicates ‘severe’ levels.

#### 2.3.9 Sleep Condition Indicator: two-item short form (SCI-02; Espie et al., 2014)

The SCI is a clinical screening tool evaluating insomnia. The two-item short-form version asks how many nights a week the respondent typically had a problem with their sleep, and to what extent has poor sleep troubled them in general, in the past month. Responses are scored from 4 to 0, with lower scores indicating poorer quality sleep.

#### 2.3.10 Specific Psychotic Experiences Questionnaire (SPEQ-H adapted; Ronald et al., 2013)

The SPEQ comprises four scales which each assess a key psychotic experience. The hallucinations scale (SPEQ-H) was used in this study. This asks respondents to rate how frequently they have recently had particular experiences (“How often do you: hear noises or sounds when there is nothing about to explain them?”) using six-point Likert scales (“0 - not at all” to “5 – daily”). This was adapted to include two further items assessing voice-hearing: ‘How often do you… hear voices saying words or sentences when there is no one around that might account for it’ and ‘…hear two or more unexplained voices talking to each other’. Higher scores indicate higher levels of hallucinatory experiences.

#### 2.3.11 Warwick-Edinburgh Mental Wellbeing Scale (WEMWBS; Tennant et al., 2007)

The WEMWBS measures psychological wellbeing (“I've been feeling cheerful”) over the past two weeks using 14 items rated from 1 “none of the time” to 5 “all of the time”. Higher scores indicate greater psychological wellbeing.

### 2.4 Statistical analysis

Analyses were conducted in R, version 3.6.1 (R Core Team, 2019). Multiple imputation for missing data was performed using the ‘mice’ package (version 3.8.0; van Buuren and Groothuis-Oudshoorn, 2020). Prior to network estimation, data were transformed to a normal distribution (using ‘gaussianize’ in ‘DAGtools’, v0.1.001l) before an undirected partial correlation network and a Bayesian inference with directed acyclic graphs (DAGs) network were estimated.

Packages ‘bootnet’ (v1.3) and ‘qgraph’ (Epskamp et al., 2012) were used to estimate and visualise the undirected (Gaussian Graphical model) network using ggmModSelect to obtain optimum model fit. Non-parametric bootstrapping (5000 bootstraps) was used to assess the accuracy and stability of the estimated network (Supplementary Material). In the final graph, positive partial correlations are shown by blue and negative correlations by red lines. The strength of the pairwise partial correlations between nodes is indicated in both cases by the weight of the edge.

The final causal graph was calculated by averaging the results of 50,000 sample DAGs, obtained by using the BiDAG package to run the partition Markov Chain Monte Carlo algorithm (Kuipers and Moffa, 2017; Kuipers et al., 2018) for 10 million iterations. Causal effects (z-scores with credible intervals; CIs) were also calculated. A credible interval may be interpreted similarly to a confidence interval, but is calculated according to the probability distribution given the data. The final graph shows edges that were present in over 50% of the 50,000 sampled DAGs and those which showed a specific direction in over 90% of cases are directed (i.e. contain an arrowhead in the probable direction of effect).

## 3 Results

Table 1 summarises the demographic details of the participant group. The majority of participants were White (68.63%), male (69.51%), outpatients (73.73%), with a diagnosis of schizophrenia (64.19%). The mean age was 41.08 years (SD = 12.24).

Table 2 shows the mean scores for each scale. High paranoia scores were common, with a third (*n* = 310; 34.37%) of participants scoring above cut-off for a persecutory delusion, and 162 (17.96%) scoring in the ‘very severe’ range of the R-GPTS Persecution scale. There were high levels of hallucinatory experiences: a third reported hearing ‘voices saying words or sentences’ daily or several times a week (*n* = 315; 34.92%), and a quarter reported ‘hear[ing] two or more unexplained voices talking to each other’ daily or several times a week (*n* = 234; 25.94%). The scores also suggest many of the participants had significantly poor sleep quality (*n* = 142, 15.74% scored 0 on the SCI-02), whilst a quarter (*n* = 225; 24.94%) rated themselves as having good sleep (the maximum score). The WEMWBS score indicates psychological wellbeing on the boundary between ‘low’ and ‘average’ ranges, with *n* = 313 (34.70%) scoring below 40, a level suggestive of depression (Warwick Medical School, 2020).

Only three participants had a recorded dissociative disorder diagnosis: ‘dissociative disorder’ (*n* = 1), ‘non-epileptic attack disorder’ (n = 1), and ‘historical diagnosis of depersonalisation’ (n = 1).

### 3.1 Prevalence of dissociation

Taking a response of ‘often’ or ‘always’ as endorsement of an item, 617 (68.40%) participants endorsed at least one item, 243 (26.94%) participants endorsed more than 25% of the items, 96 (10.64%) participants endorsed over 50%, and 29 (3.22%) endorsed over 75% of the items. A total of 285 (31.60%) participants did not endorse any ČEFSA items. Table 3 shows the response and endorsement rates of each item.

**Table 3:** Response and endorsement rates for each Černis Felt Sense of Anomaly scale item.

		% of participant group (n = 902)					
		Never	Rarely	Sometimes	Often	Always	Item endorsed (i.e. often or always)
1.	I feel like a stranger to myself.	42.90	16.74	22.62	9.31	8.43	17.74
2.	I feel detached from my physical body (or parts of it).	52.22	14.63	18.18	10.09	4.88	14.97
3.	Places that I know seem unfamiliar.	52.00	18.85	15.63	8.65	4.88	13.53
4.	I don't fully experience emotions.	32.59	17.29	24.61	17.07	8.43	25.50
5.	I feel disconnected from the world around me.	31.71	15.19	26.94	16.19	9.98	26.16
6.	I'm absorbed in my own world and don't notice what is happening around me.	31.04	17.74	25.17	16.63	9.42	26.05
7.	I feel like other people aren't real.	57.65	15.63	14.52	8.20	3.99	12.20
8.	My personality changes seemingly at random.	39.58	17.41	23.06	11.97	7.98	19.96
9.	My body (or parts of it) feels like it doesn't belong to me.	62.75	13.97	12.42	6.21	4.66	10.86
10.	Familiar sights, smells (etc.) feel unfamiliar to me.	58.54	17.74	14.08	6.32	3.33	9.65
11.	I can't feel emotions.	45.34	17.96	18.29	11.20	7.21	18.40
12.	I feel disconnected from other people.	30.16	15.74	27.38	18.18	8.54	26.72
13.	I find myself drifting off into my own world when I'm with others.	25.83	14.08	28.49	18.18	13.41	31.60
14.	The world seems like it is fake.	47.45	13.19	19.84	10.42	9.09	19.51
15.	I feel like I don't have a personality.	49.00	16.41	18.18	9.65	6.76	16.41
16.	My body (or parts of it) feels unreal or strange.	53.44	16.63	17.85	8.54	3.55	12.08
17.	People around me seem different or altered.	41.57	16.96	24.94	10.86	5.65	16.52
18.	I feel detached from my emotions.	36.81	19.40	24.06	12.53	7.21	19.73
19.	I feel as if I'm experiencing life from very far away.	43.57	14.19	23.50	11.64	7.10	18.74
20.	I don't notice how much time passes.	31.37	17.63	24.17	16.96	9.87	26.83
21.	The world around me seems unreal.	44.35	16.30	20.40	11.97	6.98	18.96
22.	I act like someone else without meaning to.	51.88	15.96	16.52	9.65	5.99	15.63
23.	My body feels like it's not under my control	49.67	16.52	18.74	8.98	6.10	15.08
24.	People I know seem unfamiliar.	50.44	18.96	19.51	7.43	3.66	11.09
25.	I feel disconnected from my emotions.	41.80	15.74	23.17	11.64	7.65	19.29
26.	The things happening around me seem unreal to me – like a dream or a movie.	39.69	16.08	23.39	11.97	8.87	20.84
27.	I lose track of my surroundings.	45.12	17.74	23.61	9.09	4.43	13.53
28.	I feel as though other people stop existing when I can't see them.	61.09	13.41	14.41	6.54	4.55	11.09
29.	I feel like I'm more than one person.	59.87	9.76	14.86	7.43	8.09	15.52
30.	My body feels numb.	52.88	13.97	20.29	7.21	5.65	12.86
31.	Things I've done many times before seem new or unfamiliar.	47.56	18.40	21.29	8.98	3.77	12.75
32.	My emotions don't seem real.	49.56	16.85	19.29	8.76	5.54	14.30
33.	I feel detached from what I'm doing.	40.35	15.08	26.50	11.75	6.32	18.07
34.	I feel like an alien or a ghost.	63.53	9.53	12.42	8.31	6.21	14.52
35.	I freeze, unable to do anything.	51.11	16.63	18.40	9.20	4.66	13.86

Of those who endorsed at least one item on the scale (*n* = 617), the mean number of items endorsed was 8.93 (SD = 8.01), and the median was 6 items. Taking endorsement of any of the five items in a factor as endorsement of that factor, the mean number of factors (i.e. different dissociation symptom types) endorsed by this subgroup was 3.94 (SD = 2.12), and the median number of factors endorsed was four. *Anomalous Experience of Emotion*, *Altered Sense of Connection*, and *Altered Sense of Agency* were the most likely to have at least one symptom being reported as occurring regularly (Supplementary Material).

### 3.2 Undirected network

Table 4 shows the partial correlations between dissociation and the other variables, extracted from the correlation matrix (Supplementary Material). FSA-dissociation was highly correlated with cognitive appraisals, and moderately correlated with responses to dissociation (RTD), paranoia, hallucinations, perseverative thinking, and affect intolerance.

**Table 4:** Correlations between dissociation and each variable (n = 902).

Variable	Pairwise partial correlation
Cognitive appraisals	0.79
Responses to dissociation	0.53
Paranoia	0.58
Hallucinations	0.63
General self-efficacy	−0.28
Perseverative thinking	0.67
Affect intolerance	0.56
Alexithymia	−0.40
Wellbeing	−0.43
Sleep quality	−0.40

**Fig. 1:**
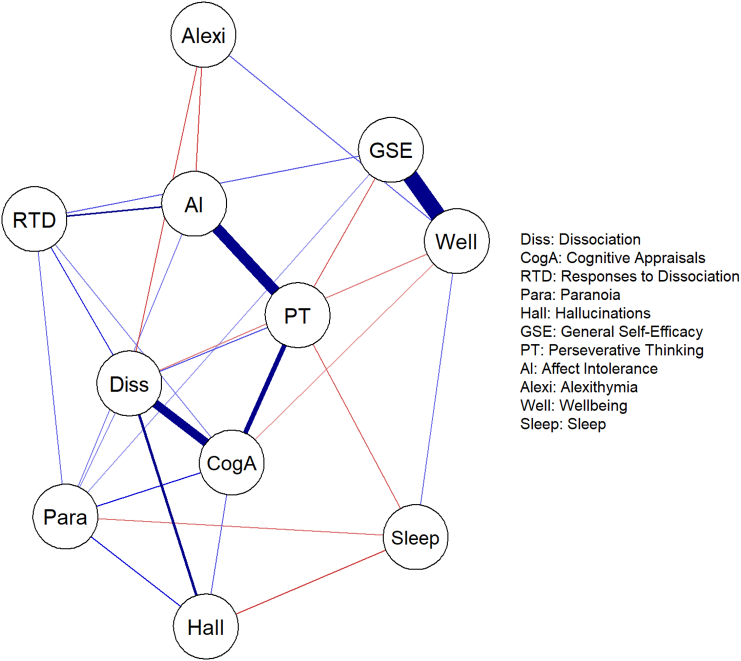
Undirected network graph showing relationships between dissociation, symptoms, and candidate maintenance mechanisms. (Red lines show negative relationships. Blue lines show positive relationships. Greater thickness and colour strength of edges indicates greater edge weight). (For interpretation of the references to colour in this figure legend, the reader is referred to the web version of this article.)

Fig. 1 shows the undirected network (full estimation details in Supplementary Material). In summary, dissociation had direct relationships with most variables, but not with affect intolerance, general self-efficacy, or sleep. Dissociation was most strongly connected to cognitive appraisals about dissociation (CAD). Like dissociation, CAD also did not have a direct edge with affect intolerance, general self-efficacy, or sleep. The network consists of many short paths, affecting the stability of the betweenness centrality estimate. However, this is above minimum recommended levels, and the stability of closeness and strength centrality estimates were good.

### 3.3 Bayesian inference with Directed Acyclic Graphs (DAGs)

**Fig. 2:**
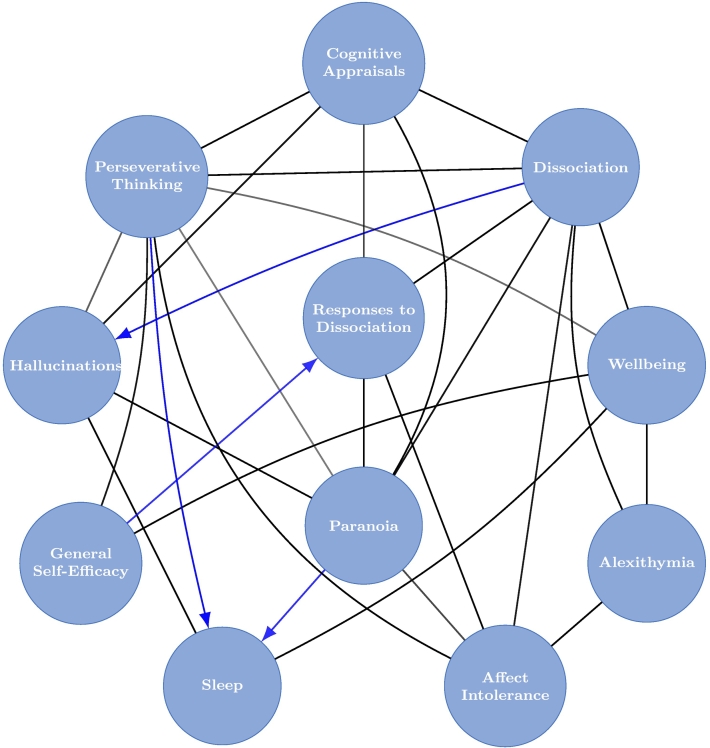
Mixed graph (both directed and undirected edges) showing relationships between the variables. Undirected lines show direct relationships present in over 50.00% of 50,000 sampled DAGs. Lines with arrowheads show the probable direction of influence if this was present in over 90.00% of 50,000 sampled DAGs.

Fig. 2 illustrates the result of the DAGs estimation. Only direct pathways (edges) present in over 50.00% of the 50,000 sampled DAGs are represented. Edges which were present *and* showed the same direction of influence in over 90.00% of the sampled DAGs are shown with an arrowhead indicating the direction of effect.

Table 5 summarises the directions of relationships between dissociation and all other variables in the network.

**Table 5:** Average causal effects between dissociation and all other variables.

Causal effects:	Pathway present (direct or indirect) %	Causal effect	90% CI	Direct edge present %	Direct causal effect	90% CI
Variable to dissociation (i.e. variable causing dissociation)^a^						
Cognitive Appraisals	15.28	0.72	0.55–0.81	100	0.64	0.49–0.79
Responses to Dissociation	17.28	0.43	0.17–0.58	100	0.29	0.15–0.55
Paranoia	13.75	0.38	0.13–0.65	99.42	0.29	0.10–0.62
Hallucinations	5.79	0.43	0.19–0.67	100	0.33	0.18–0.66
General self-efficacy	30.19	−0.22	−0.31 - 0.024	28.13	−0.046	−0.26–0.00
Perseverative thinking	18.28	0.52	0.19–0.70	99.89	0.37	0.14–0.64
Affect intolerance	22.28	0.41	0.073–0.61	94.80	0.34	0.00–0.59
Alexithymia	24.06	−0.33	−0.44 to −0.19	100	−0.29	−0.42 to −0.17
Wellbeing	43.06	−0.35	−0.47 to −0.12	100	−0.29	−0.45 to −0.10
Sleep	2.52	−0.20	−0.46 to −0.016	35.71	−0.072	−0.38–0.00

Dissociation to variable (i.e. dissociation causing variable)^a^						
Cognitive appraisals	84.72	0.69	0.47–0.79	100	0.51	0.41–0.74
Responses to dissociation	82.72	0.49	0.30–0.61	100	0.34	0.22–0.54
Paranoia	86.25	0.48	0.22–0.60	93.40	0.25	0.00–0.52
Hallucinations	94.21	0.53	0.35–0.63	100	0.35	0.25–0.47
General self-efficacy	50.75	−0.23	−0.31 to −0.025	9.84	−0.017	−0.18–0.00
Perseverative thinking	81.72	0.53	0.21–0.69	98.09	0.29	0.094–0.50
Affect intolerance	77.18	0.46	0.11–0.61	90.50	0.32	0.00–0.59
Alexithymia	75.94	−0.33	−0.44 to −0.22	100	−0.27	−0.40 to −0.17
Wellbeing	59.90	−0.36	−0.46 to −0.20	99.65	−0.30	−0.44 to −0.15
Sleep	96.86	−0.24	−0.36 to −0.073	8.75	−0.011	−0.097–0.00


Key:	
‘Pathway present’	The proportion of sampled DAGs which found this pathway.
‘Causal effect’	Average total causal effect when that pathway was present.
‘Direct edge present’	The proportion of DAGs that found direct pathways of those where some pathway was found to be present.
‘Direct causal effect’	Average total causal effect of the direct pathways.
CI	Credible interval.

a Percentages to 2 decimal places, causal effects & credible intervals to 2 significant figures.

The results can be interpreted as in the following example: a pathway (direct or indirect) *from* paranoia *to* dissociation was present in 13.75% of the 50,000 DAGs sampled. The average strength of the effect of paranoia on dissociation within these graphs was 0.38 (with a 90% credible interval 0.13–0.65). In 99.42% of the 13.75% of sampled DAGs a direct edge from paranoia to dissociation was present, and this had an average strength of 0.29 (90% CI = 0.10–0.62). In the opposite direction, from dissociation to paranoia, there was a pathway present in 86.25% of the 50,000 sampled graphs, with an average strength of 0.48 (CI = 0.22–0.60). Of these, 93.40% contained a direct edge from dissociation to paranoia. The average strength of this was 0.25 (CI = 0.00–0.52). Therefore, for the relationship between paranoia and dissociation, there is a non-significant indication (found in more than 50% but less than 90% of sampled DAGs) that dissociation has a moderate influence on paranoia. The direction of influence whereby paranoia affects dissociation was found in fewer than 50% of sampled DAGs, indicating a low probability of this direction of effect.

The only edge between dissociation and another variable reaching the 90% threshold for inferring probable direction of effect was with hallucinations. Here, it was probable that dissociation is a causal factor for hallucinations, with moderate effect size (0.53, CI = 0.21–0.69).

Sleep and psychological wellbeing both had negative correlations with dissociation. For sleep, the direction of influence reached the 90% threshold for inferring dissociation was a probable cause of poor sleep. However, this effect was largely indirect, with fewer than 10% of the sampled DAGs with a pathway between dissociation and sleep containing the direct pathway. Inspection revealed that pathways between dissociation and sleep commonly (i.e. >50% of the 96.86% of DAGs) included perseverative thinking, hallucinations, paranoia, cognitive appraisals, and affect intolerance. In contrast, the result for wellbeing was ambiguous in terms of direction, but the vast majority of effect was via the direct pathway.

As in the undirected model, dissociation had direct relationships (over the 50% threshold) with all potential maintenance mechanisms except general self-efficacy and sleep. It is a point of difference between the two networks that the DAGs analysis found a direct relationship between dissociation and affect intolerance, where none was found in the undirected network, although it should be noted that this was only marginally above the 50% threshold (50.75% of 50,000 DAGs).

In terms of likely direction of influence, no relationship between dissociation and a candidate maintenance factor reached the 90% threshold. There were trends towards dissociation being a causal influence for nearly all mechanism factors except general self-efficacy, and particularly in the case of cognitive appraisals, cognitive-behavioural responses to dissociation, and perseverative thinking which were influenced by dissociation in over 80% of sampled DAGs. The correlations between dissociation and general self-efficacy and alexithymia were negative.

## 4 Discussion

This study is the largest exploration of dissociation in psychosis. The patient group had relatively high levels of paranoia and hallucinations, with a third scoring above cut-off for a persecutory delusion and a third hearing voices daily or several times a week. In this context FSA-type dissociation was also common, with two-thirds of the group experiencing at least one such dissociative experience frequently over the past fortnight. Of those who did report dissociation, an average of nine dissociative experiences were reported, comprising an average of four different types. The most common type was altered sensations of agency (e.g. “*I lose track of my surroundings*”). The next most common were experiences of an altered sense of connection (“*I feel as if I'm experiencing life from very far away*”) and anomalous experience of emotion (“*I don't fully experience emotions*”). These findings are in contrast to the total of three co-morbid dissociative disorder diagnoses recorded in this group's clinical notes, which itself is far lower than previous estimates (Renard et al., 2017) and illustrates the extent to which dissociative phenomena may be overlooked in routine clinical practice. The results are also strongly suggestive that FSA-dissociation is a cause of hallucinations and paranoia.

Dissociation had direct relationships with cognitive appraisals, cognitive-behavioural responses to dissociation, perseverative thinking, and low alexithymia (but not to sleep). Our view is that these results suggest dissociation may be maintained by a process related to affect sensitivity and an interacting process reinforcing dissociation via cognitive and behavioural responses to the experience. Direct relationships between dissociation and affect intolerance and alexithymia (i.e. self-rated detection of emotion) indicate an affect sensitivity process may be relevant, whilst direct and close inter-connections found between dissociation, cognitive appraisals, cognitive-behavioural responses to dissociation, and perseverative thinking are consistent with a general cognitive-behavioural perspective. Potentially linking the two hypothesised processes, perseverative thinking (a cognitive-behavioural process) had a direct relationship with affect intolerance (a key factor in the hypothesised affect sensitivity process). General self-efficacy was directly linked to perseverative thinking and responses to dissociation, suggesting this factor might indirectly influence the maintenance of dissociation.

It is important to note that, as expected, whilst these relationships were robust, the results of the DAGs analysis do not unambiguously find that these potential maintenance factors causally influence dissociation. In all cases, the relationship between these variables and dissociation did not reach the 90% threshold for drawing conclusions about direction of influence. Indeed, the majority of these relationships (with the exception of general self-efficacy) showed strong trends towards dissociation having causal influence. Unlike relationships between dissociation and psychotic symptoms, the question of how to interpret ambiguous directions of influence between potential maintenance factors and dissociation is not as clearly indicated by the existing literature. For example, it may be that there are sufficient individual differences in the response to dissociation (or which mechanisms are influential in its maintenance) that heterogeneity presents as a weaker overall causal effect from each mechanism to dissociation at a group level.

It is arguably a limitation of the current study that only two (positive) psychotic symptoms were measured. As psychosis research and intervention strategies increasingly take a symptom-specific approach (Freeman et al., 2021), it would be valuable to explore relationships between dissociation and a broader range of symptoms, including negative psychotic symptoms. It could also be helpful to explore such associations at the level of individual FSA-dissociation factors, as different domains or types of FSA may have differential patterns of association with psychotic symptoms. For example, Humpston et al. (2020) report differential associations between depersonalisation and derealisation and first rank symptoms of schizophrenia over a twenty-year longitudinal study.

## 5 Conclusions

The implication of models proposing that dissociation causes psychotic experiences is that reducing dissociation may therefore alleviate them. Recent publications reflect increasing clinical interest in addressing this hypothesis. For example, Farrelly et al. (2016) outline a protocol for a brief CBT intervention for depersonalisation and derealisation in psychosis, and McCartney et al. (2019) and Varese et al. (2020) demonstrate there is potentially a significant clinical benefit from targeting dissociation (as well as voices and trauma) in patients experiencing psychosis with a history of interpersonal trauma. The development of effective targeted psychological interventions for dissociation in psychosis will depend upon a robust understanding of its causal mechanisms. In this study, we have demonstrated that FSA-dissociative experiences in psychosis occur in the context of a closely-bound network of psychological factors including cognitive-behavioural and affect-sensitivity processes. A logical next step in this work would be to carry out experimental studies testing the most clinically effective way to intervene in this interconnected network, thereby potentially disrupting the maintenance of dissociation.

## Data availability statement

The authors do not have participant consent to share the data collected in this study.

## Funding statement

The work was supported by the 10.13039/100010269Wellcome Trust via a Clinical Doctoral Fellowship to EČ (grant number 102176/B/13/Z). DF was supported during this work by an 10.13039/501100000272NIHR Research Professorship (NIHR-RP-2014-05-003) and is an NIHR Senior Investigator. AE is funded by the 10.13039/100010269Wellcome Trust (200796) and supported by the 10.13039/501100013373NIHR Oxford Health Biomedical Research Centre and a NIHR Senior Investigator Award. The views expressed are those of the authors and not necessarily those of the National Health Service, NIHR, or Department of Health.

The funder had no role in the study design, collection, analysis or interpretation of data, writing of the report, or in the decision to submit the article for publication.

## Declaration of competing interest

The authors declare no conflicts of interest.

## Appendix A Supplementary data

Further Analysis Image 1
