# Supplementary material for: Dissociation in patients with non-affective psychosis: Prevalence, symptom associations, and maintenance factors
Source: Schizophr Res. 2022 Jan;239:11–8. doi: 10.1016/j.schres.2021.11.008 (PMC8765411; doi:10.1016/j.schres.2021.11.008)
Supplement: Supplementary file 1 — Further Analysis [file mmc1.docx]

Supplementary Material – Endorsement of ČEFSA items

***Figure 1.*** A histogram displaying the distribution of ČEFSA total scores in the participant group.


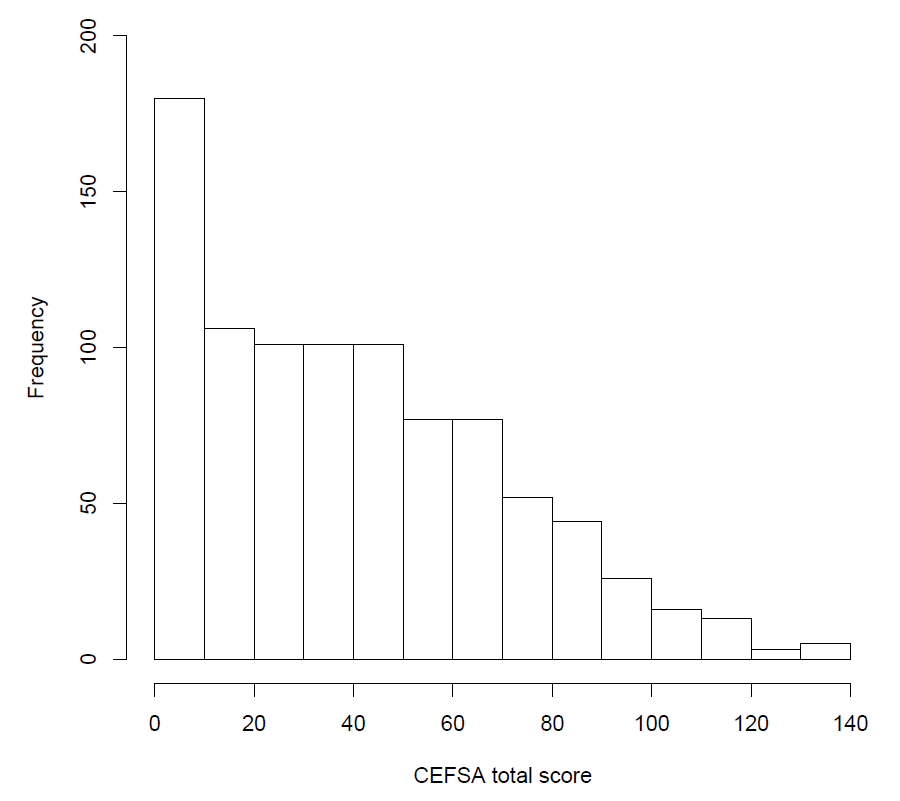


Skewness co-efficient of ČEFSA = 0.621, indicating moderate skew.

This was consistent with the moderate skewness of the psychotic symptom scores in this group (paranoia=0.544; hallucinations=0.730).

| ***Table 1.*** Rates of endorsement for the ČEFSA (whole scale and factors). | | | | |
| --- | --- | --- | --- | --- |
|  | Score  *Mean (SD)* | No. of items endorsed  *Mean (SD)* | No. patients endorsing 0 items  *N (%)* | No. patients endorsing ≥1 item  *N (%)* |
| ČEFSA total | 40.56 (30.59) | 6.11 (7.81) | 285 (31.60) | 617 (68.40) |
| Anomalous Experience of the Self | 5.60 (5.02) | 0.85 (1.33) | 547 (60.64) | 355 (39.36) |
| Anomalous Experience of the Body | 4.73 (4.81) | 0.66 (1.21) | 620 (68.74) | 282 (31.26) |
| Altered Sense of Familiarity | 4.94 (4.65) | 0.97 (1.53) | 620 (68.74) | 282 (31.26) |
| Anomalous Experience of Emotion | 6.34 (5.38) | 1.11 (1.53) | 558 (61.86) | 344 (45.90) |
| Altered Sense of Connection | 7.04 (5.33) | 1.12 (1.44) | 488 (54.10) | 414 (45.90) |
| Altered Sense of Agency | 7.01 (5.03) | 0.85 (1.33) | 460 (51.00) | 442 (49.00) |
| Altered Sense of Reality | 4.91 (4.95) | 0.76 (1.28) | 593 (65.74) | 309 (34.26) |

***Figure 2.*** Graph showing the rates of endorsement of individual ČEFSA items.


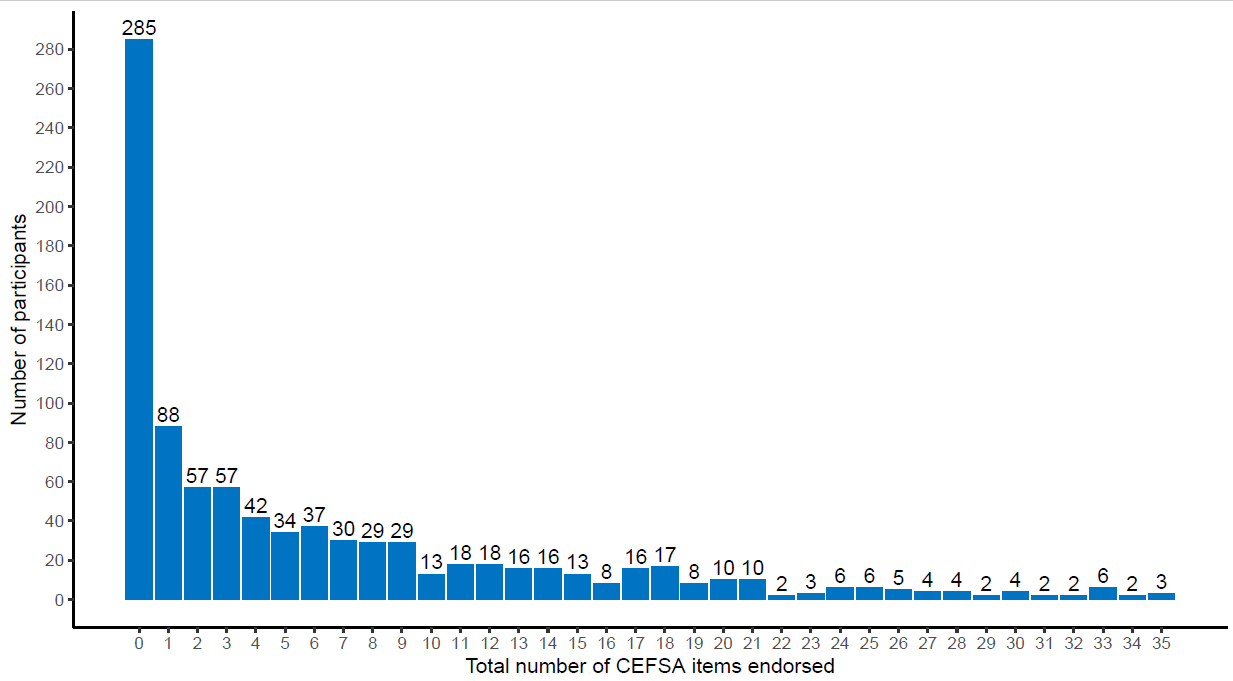


Supplementary Material – Network estimation

Correlation matrix:

|  | Diss | CogA | RTD | Para | Hall | GSE | PT | AI | Alexi | Well | Sleep |
| --- | --- | --- | --- | --- | --- | --- | --- | --- | --- | --- | --- |
| Diss | - | 0.79 | 0.53 | 0.58 | 0.63 | -0.28 | 0.67 | 0.56 | -0.40 | -0.43 | -0.40 |
| CogA | 0.79 | - | 0.52 | 0.60 | 0.59 | -0.29 | 0.72 | 0.59 | -0.35 | -0.42 | -0.38 |
| RTD | 0.53 | 0.52 | - | 0.45 | 0.41 | -0.02 | 0.50 | 0.52 | -0.24 | -0.17 | -0.26 |
| Para | 0.58 | 0.60 | 0.45 | - | 0.55 | -0.13 | 0.52 | 0.45 | -0.26 | -0.26 | -0.36 |
| Hall | 0.63 | 0.59 | 0.41 | 0.55 | - | -0.22 | 0.52 | 0.42 | -0.28 | -0.31 | -0.39 |
| GSE | -0.28 | -0.29 | -0.02 | -0.13 | -0.22 | - | -0.34 | -0.18 | 0.23 | 0.65 | 0.17 |
| PT | 0.67 | 0.72 | 0.50 | 0.52 | 0.52 | -0.34 | - | 0.70 | -0.36 | -0.45 | -0.42 |
| AI | 0.56 | 0.59 | 0.52 | 0.45 | 0.42 | -0.18 | 0.70 | - | -0.36 | -0.29 | -0.34 |
| Alexi | -0.40 | -0.35 | -0.24 | -0.26 | -0.28 | 0.23 | -0.36 | -0.36 | - | 0.32 | 0.21 |
| Well | -0.43 | -0.42 | -0.17 | -0.26 | -0.31 | 0.65 | -0.45 | -0.29 | 0.32 | - | 0.32 |
| Sleep | -0.40 | -0.38 | -0.26 | -0.36 | -0.39 | 0.17 | -0.42 | -0.34 | 0.21 | 0.32 | - |

Key:

| *Diss* | Dissociation |
| --- | --- |
| *CogA* | Cognitive Appraisals |
| *RTD* | Responses to Dissociation |
| *Para* | Paranoia |
| *Hall* | Hallucinations |
| *GSE* | General Self Efficacy |
| *PT* | Perseverative Thinking |
| *AI* | Affect Intolerance |
| *Alexi* | Alexithymia |
| *Well* | Wellbeing |
| *Sleep* | Sleep quality |

**Undirected network**

Bootstrapped confidence intervals (CIs) for each edge, calculated using non-parametric bootstrapping (5000 bootstraps) using bootnet (v1.3), illustrate the accuracy of the connections in the estimated undirected network (Figure 1). Overall, the network appears to be estimated with good accuracy. The edge between general self-efficacy and wellbeing was very strong, and did not overlap with the CIs for any other edge.


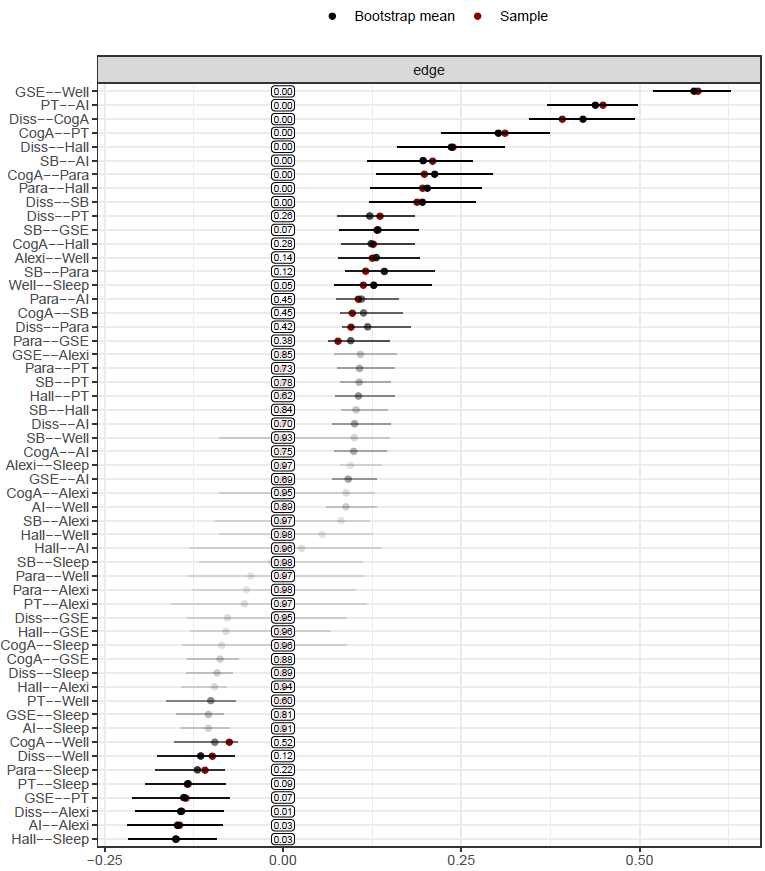
***Figure 1.*** Showing sample edge-weights and bootstrapped confidence intervals of edge-weights obtained via non-parametric bootstrapping (5000 bootstraps) for all edges in the undirected network

Table 1 shows the edge-weights and their bootstrapped confidence intervals for edges between dissociation and all other variables, and Figure 2 shows the edge-weight difference tests. These show that the strongest edge with dissociation was between cognitive appraisals and dissociation, and that this was significantly stronger than any other edge with dissociation. The second strongest was with hallucinations, which was not significantly stronger than the edge between dissociation and safety behaviours (nor the edge between hallucinations and paranoia).

| ***Table 1.*** *E*dge-weights and their bootstrapped confidence intervals for edges between dissociation and each other variable (3 s.f.) | | |
| --- | --- | --- |
| **Variable** | **Edge-weight** | **C.I.** |
| Cognitive Appraisals | 0.392 | 0.316 – 0.468 |
| Safety Behaviours | 0.188 | 0.108 – 0.268 |
| Paranoia | 0.0955 | -0.0283 – 0.219 |
| Hallucinations | 0.239 | 0.163 – 0.315 |
| General Self-Efficacy | (no edge) | -0.0394 – 0.0394 |
| Perseverative Thinking | 0.136 | 0.0192 – 0.253 |
| Affect Intolerance | (no edge) | -0.0946 – 0.0946 |
| Alexithymia | -0.142 | -0.211 – -0.0726 |
| Wellbeing | -0.0990 | -0.192 – -0.00581 |
| Sleep | (no edge) | -0.0604 – 0.0604 |

***Figure 2*.** Differences between edge-weights: statistically significant differences indicated by a black square, non-significant differences by a grey square

| ***Key:*** | |
| --- | --- |
| *AI* | *Affect intolerance* |
| *Alexi* | *Alexithymia* |
| *CogA* | *Cognitive appraisals* |
| *Dis* | *Dissociation* |
| *GSE* | *General self-efficacy* |
| *Hall* | *Hallucinations* |
| *Para* | *Paranoia* |
| *PT* | *Perseverative thinking* |
| *SB* | *Safety behaviours* |
| *Sleep* | *Sleep* |
| *Well* | *Wellbeing* |


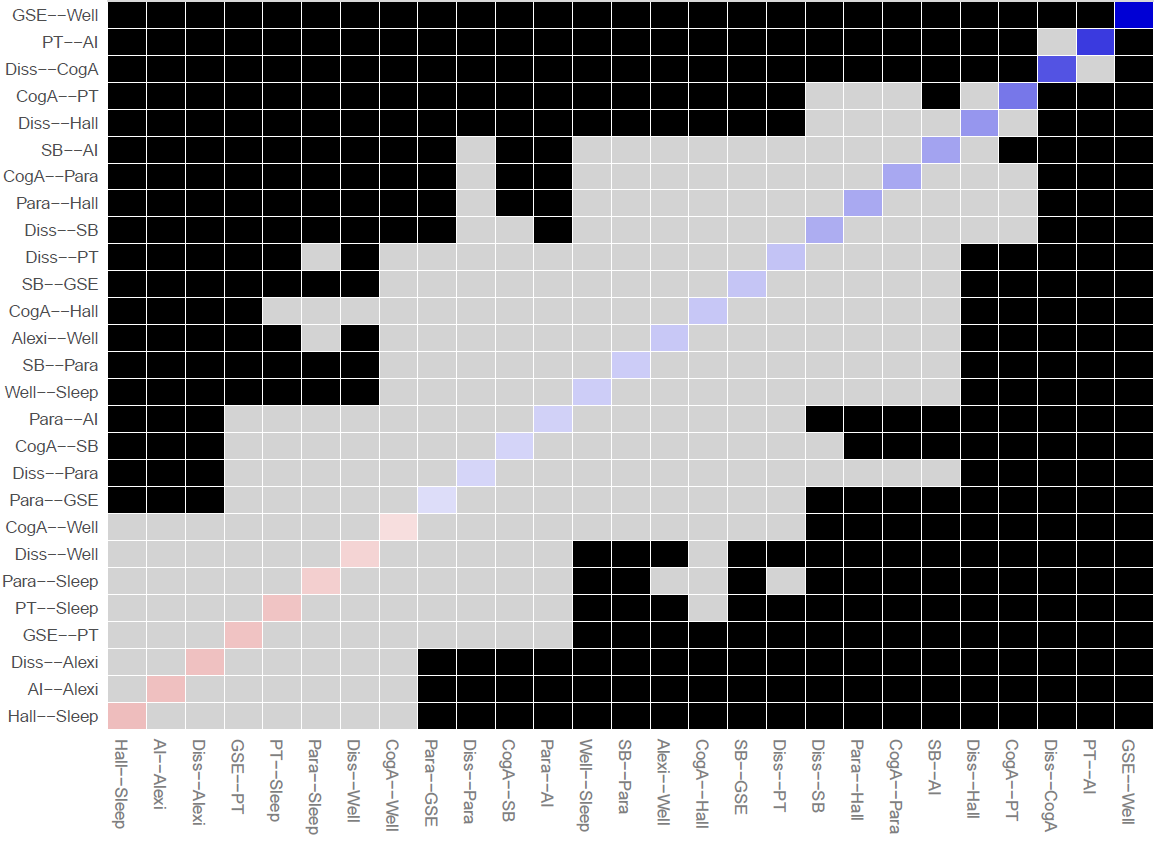


Chapter 7

209

Centrality estimates (strength, closeness and betweenness) were calculated for all variables. The results are shown in Figure 3 and Table 2. The results of difference tests for all centrality estimates for all variables are shown in Figure 4.

***Figure 3.*** Showing centrality scores for all variables in the network


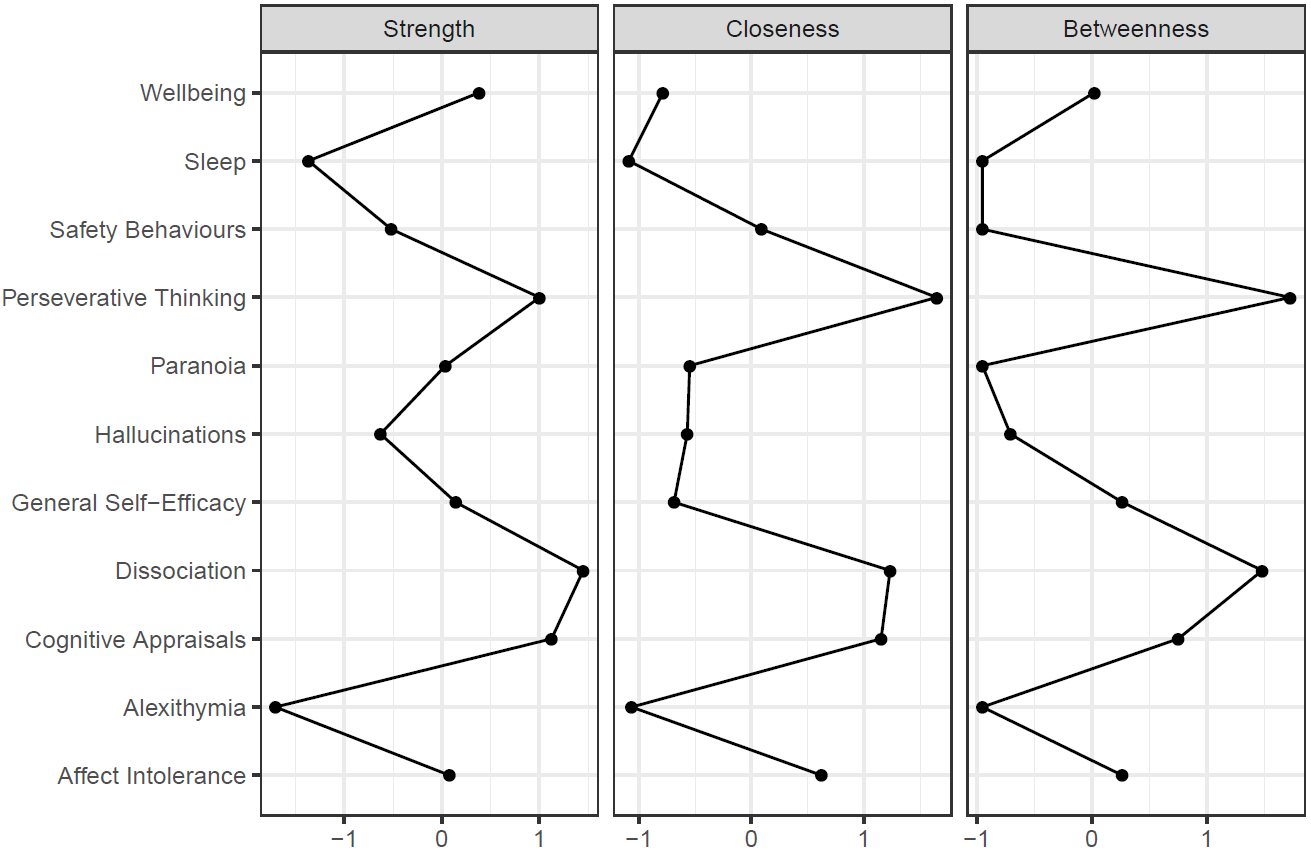


| ***Table 2.*** Centrality measures for all variables. (Degree centrality and closeness to 3 s.f.) | | | |  |
| --- | --- | --- | --- | --- |
| **Variable** | **Strength (Degree)** | **Closeness** | **Betweenness** | |
| Dissociation | 1.29 | 0.0137 | 20 | |
| Cognitive Appraisals | 1.20 | 0.0135 | 14 | |
| Safety Behaviours | 0.743 | 0.0116 | 0 | |
| Paranoia | 0.898 | 0.0105 | 0 | |
| Hallucinations | 0.712 | 0.0104 | 2 | |
| General Self-Efficacy | 0.927 | 0.0102 | 10 | |
| Perseverative Thinking | 1.17 | 0.0144 | 22 | |
| Affect Intolerance | 0.909 | 0.0126 | 10 | |
| Alexithymia | 0.412 | 0.00955 | 0 | |
| Wellbeing | 0.994 | 0.0100 | 8 | |
| Sleep | 0.506 | 0.00950 | 0 | |

| ***Figure 4.*** Differences between centrality estimates (statistically significant differences indicated by a black square, non-significant differences by a grey square) | | |
| --- | --- | --- |
| *Panel 4a: Strength (degree centrality)*  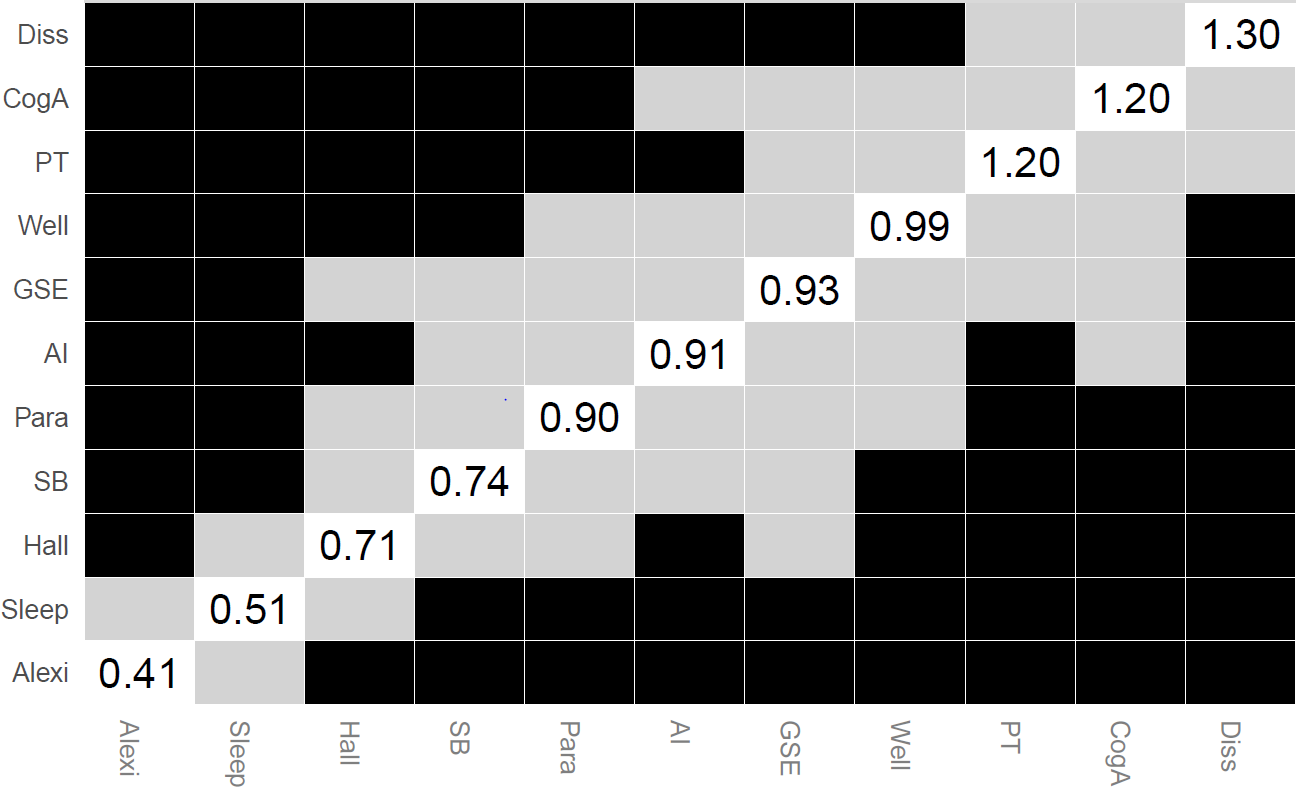 | *Panel 4c: Betweenness*  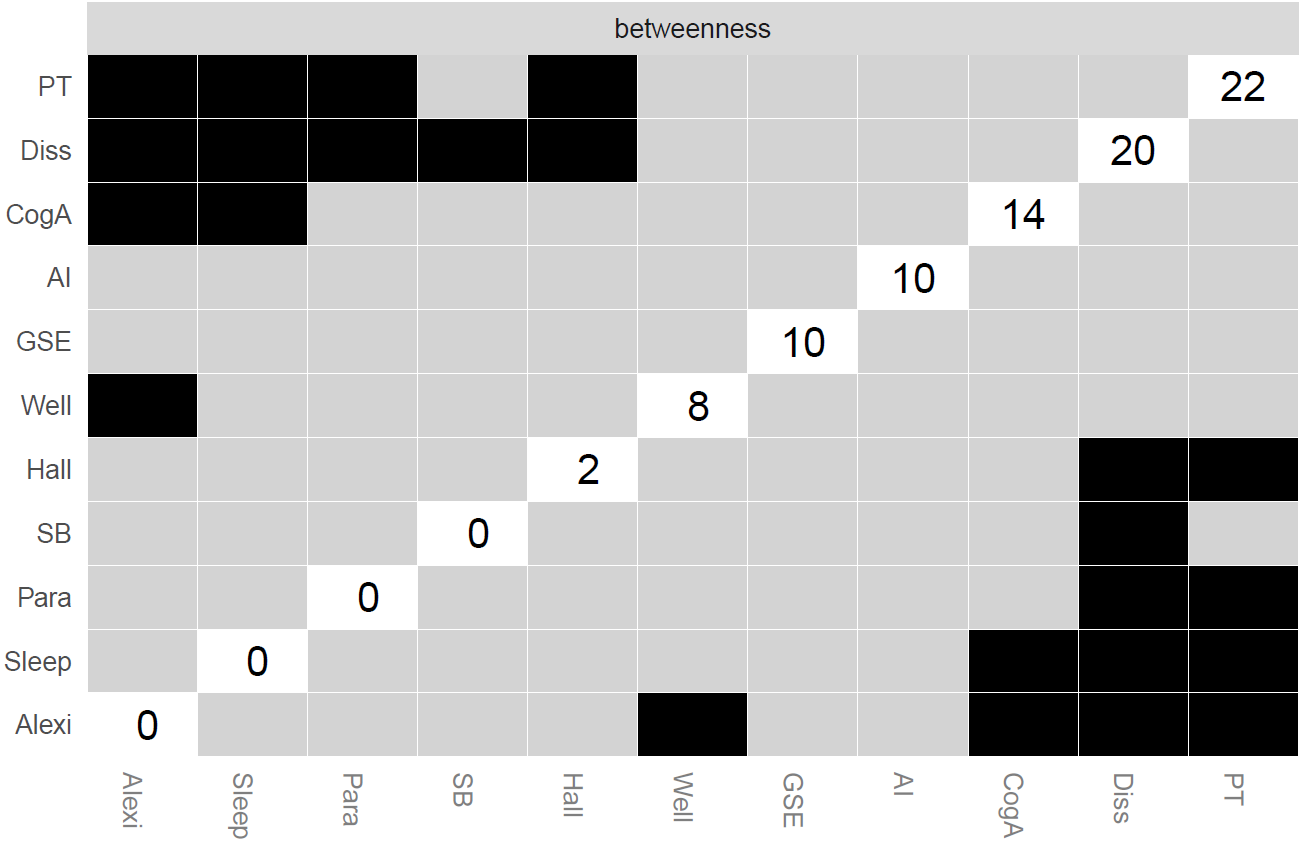 | |
| *Panel 4b: Closeness*  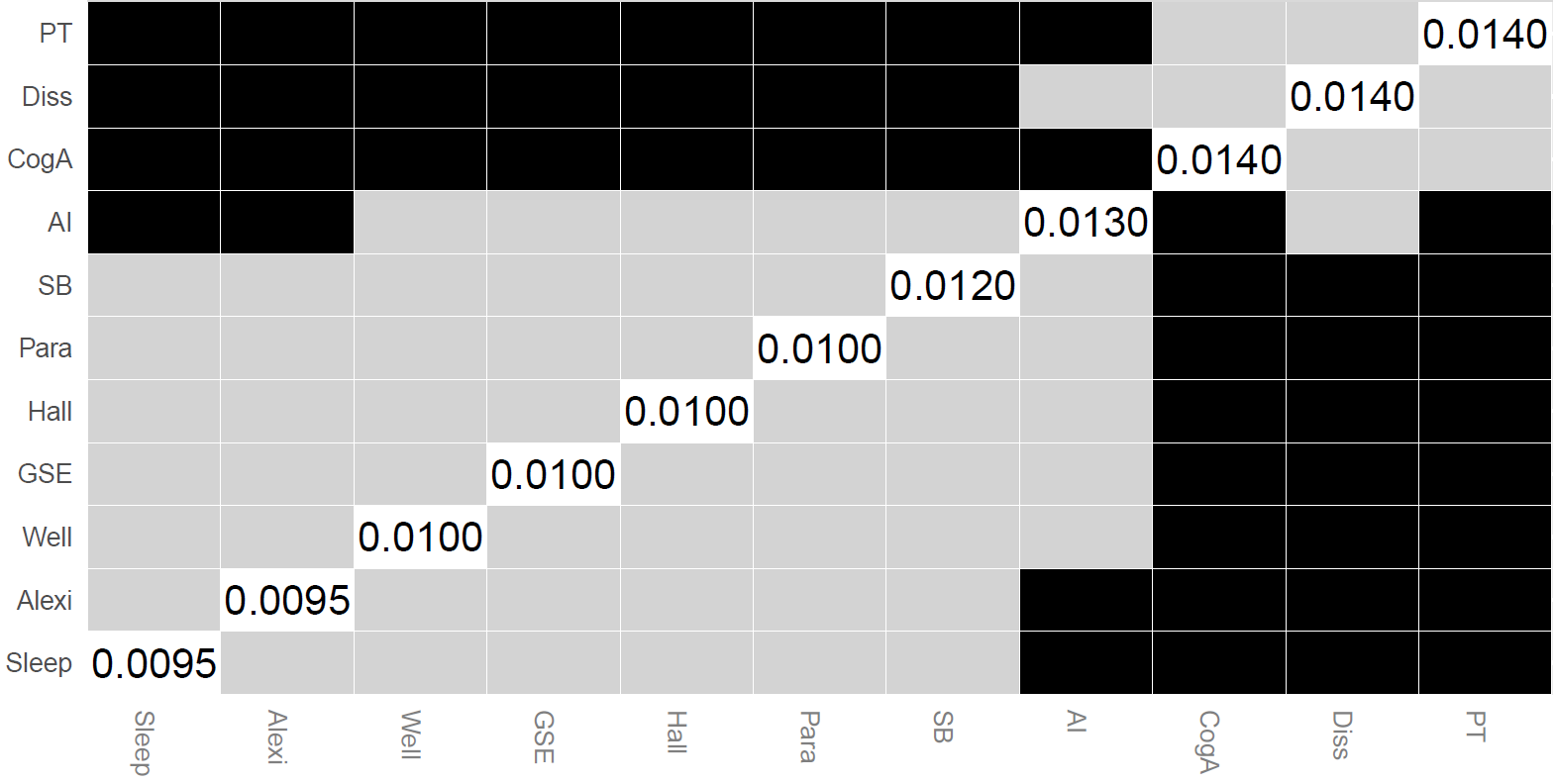 | | \| ***Key:*** \| \| \| --- \| --- \| \| *AI* \| *Affect intolerance* \| \| *Alexi* \| *Alexithymia* \| \| *CogA* \| *Cognitive appraisals* \| \| *Dis* \| *Dissociation* \| \| *GSE* \| *General self-efficacy* \| \| *Hall* \| *Hallucinations* \| \| *Para* \| *Paranoia* \| \| *PT* \| *Perseverative thinking* \| \| *SB* \| *Safety behaviours* \| \| *Sleep* \| *Sleep quality* \| \| *Well* \| *Wellbeing* \| |

The results of the centrality estimates indicate that dissociation has the highest node strength in the network. Difference tests found that its node strength was not significantly higher than that of cognitive appraisals or perseverative thinking. Perseverative thinking had the highest closeness centrality in the network, but this was not significantly higher than those of dissociation or cognitive appraisals. A similar pattern was found in the betweenness scores: perseverative thinking had the highest betweenness centrality, but this was not significantly greater than those for dissociation, cognitive appraisals, affect intolerance, general self-efficacy, wellbeing, or safety behaviours. Overall, these results suggest that dissociation, cognitive appraisals, and perseverative thinking are particularly highly connected within the network. The high levels of betweenness signify many short pathways between nodes.

Finally, the stability of centrality estimates was calculated using case-dropping subset bootstrapping (Figure 5). This indicated that the stability for all three centrality measures was good. Correlation stability coefficients for betweenness was 0.36, closeness 0.75, and strength 0.75, which meet the recommendation that coefficients should preferably be above 0.50, and not below 0.25.

***Figure 5.*** Showing the results of the case-drop subset bootstrapping


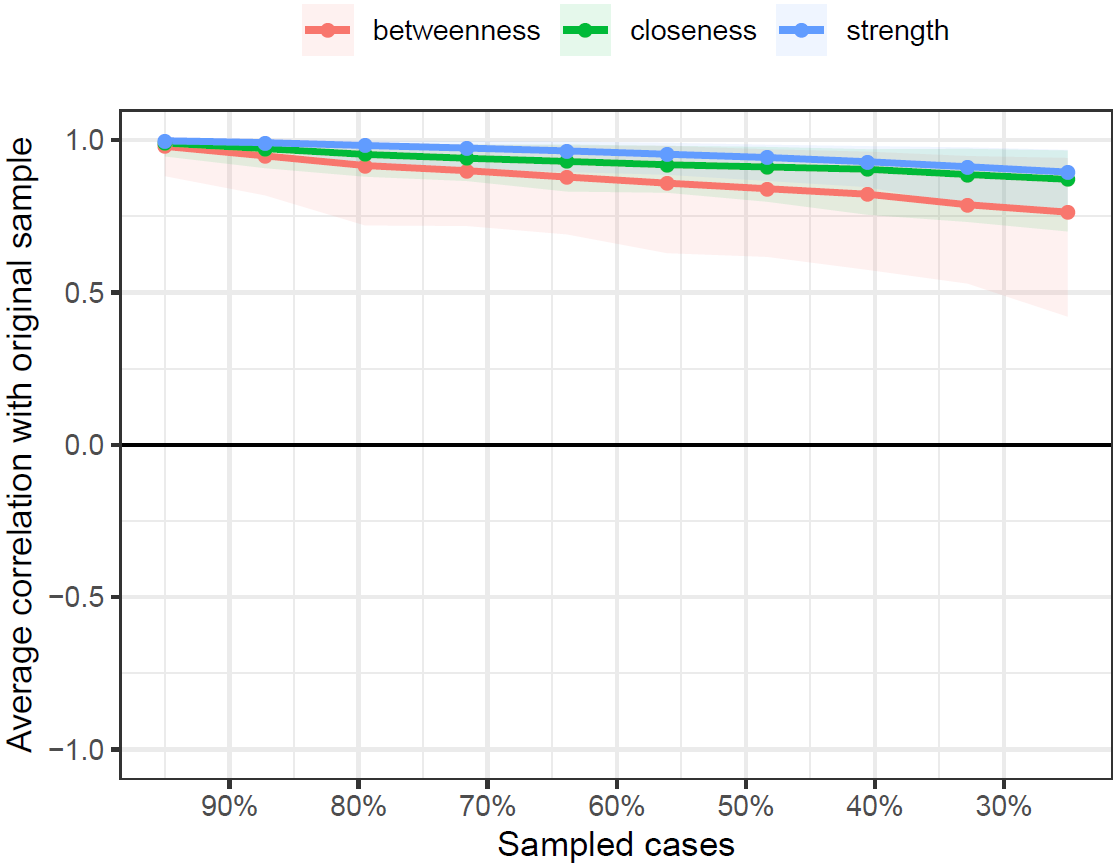


Supplementary Material – Network analysis code

####load packages####

library(rje)

library(purrr)

library(ggplot2)

library(graph)

library(Rgraphviz)

library(RBGL)

library(readxl)

library(BiDAG)

library(beepr)

library(devtools)

library(DAGtools)

####UNDIRECTED / GAUSSIAN GRAPHICAL####

#select measure total scores

dat1 <- dat[,c("CEFSA_total", "CAD_total", "RTD_total", "GPTS_total", "SPEQ.H_total", "GSE_total", "PTQ_total", "AIS_total", "Alexi_total", "WEMWBS_total", "SCI_total")]

names(dat)[1] <- "Diss"

names(dat)[2] <- "CogA"

names(dat)[3] <- "SB"

names(dat)[4] <- "Para"

names(dat)[5] <- "Hall"

names(dat)[6] <- "GSE"

names(dat)[7] <- "PT"

names(dat)[8] <- "AI"

names(dat)[9] <- "Alexi"

names(dat)[10] <- "Well"

names(dat1)[11] <- "Sleep"

## Correlations ##

cor(dat, use = "pairwise.complete.obs")

cor <- data.frame(cor(dat, use="pairwise.complete.obs"))

write.csv(cor, "correlations.csv", row.names = F)

## Network analysis ##

# Names of variables - create vector of names for the legend

names <- c("Dissociation", "Cognitive Appraisals","Safety Behaviours","Paranoia","Hallucinations","General Self-Efficacy","Perseverative Thinking","Affect Intolerance","Alexithymia","Wellbeing","Sleep")

## estimate network using ggmmodselect

dat <- gaussianize(dat)

glasso <- estimateNetwork(dat, default = "ggmModSelect")

pdf("Fig 1 – undirected network.pdf", width = 9, height = 5)

plot(glasso, cut = 0.2, legend = T, nodeNames = names, legend.cex = 0.40)

dev.off()

## Bootstrap to get CIs of edge weights ##

set.seed(123)

Boot_cis <- bootnet(glasso, nBoots = 5000, default = "ggmModSelect", nCores = 8)

saveRDS(Boot_cis, "Boot_CIs FINAL - PDirec off.RDS")

# Figure of confidence intervals of the edge weights

pdf("Figure 1 - Confidence intervals - PDirec off.pdf", width = 7, height = 8)

plot(Boot_cis, order = "sample", plot = "interval", split0 = TRUE)

dev.off()

## Get the edge weights with confidence intervals

# create dataframe of CIs

ci <- as.data.frame(summary(Boot_cis))

# isolate all edges with dissociation

CogA <- ci[ci$id=="Diss--CogA",]

SB <- ci[ci$id=="Diss--SB",]

Para <- ci[ci$id=="Diss--Para",]

Hall <- ci[ci$id=="Diss--Hall",]

GSE <- ci[ci$id=="Diss--GSE",]

PT <- ci[ci$id=="Diss--PT",]

AI <- ci[ci$id=="Diss--AI",]

Alexi <- ci[ci$id=="Diss--Alexi",]

Well <- ci[ci$id=="Diss--Well",]

Sleep <- ci[ci$id=="Diss--Sleep",]

# vector of edges

edges <- c(CogA$sample, SB$sample, Para$sample, Hall$sample, GSE$sample, PT$sample, AI$sample, Alexi$sample, Well$sample, Sleep$sample)

# vector of lower CIs

lowerCI <- c(CogA$CIlower, SB$CIlower, Para$CIlower, Hall$CIlower, GSE$CIlower, PT$CIlower, AI$CIlower, Alexi$CIlower, Well$CIlower, Sleep$CIlower)

# vector of lower CIs

upperCI <- c(CogA$CIupper, SB$CIupper, Para$CIupper, Hall$CIupper, GSE$CIupper, PT$CIupper, AI$CIupper, Alexi$CIupper, Well$CIupper, Sleep$CIupper)

# create table of edges and confidence intervals

table <- tibble(Variables = names[-1]) # using the name vector from earlier, but deleted paranoia so just the other variable names -

table2 <- table %>%

mutate(edges = edges, lowerCI = lowerCI, upperCI = upperCI)

write.csv(table2, "Undirected Edge weights- PDirec off.csv", row.names = F)

# plot of edge weight differences

pdf("figure 2 Edge differences.pdf", width = 7, height = 8 * 2/3)

plot(Boot_cis, statistics = "edge", order = 'sample', plot = "difference", onlyNonZero = T)

dev.off()

## centrality indices ##

cent <- centrality(glasso)

round(cent$OutDegree,4)

round(cent$Closeness,5)

cent$Betweenness

# centrality plot

pdf("Figure 3 centrality summary.pdf", width = 7, height = 7 * 2/3)

centralityPlot(glasso, include = c("Strength","Closeness","Betweenness"), theme_bw = TRUE, labels = names)

dev.off()

## Bootstrap to test stability of centrality estimates##

set.seed(123)

Boot_centrality <- bootnet(glasso, nBoots = 5000, default = "ggmModSelect", nCores = 8, statistics=c("strength", "closeness", "betweenness"), order = 'sample')

saveRDS(Boot_centrality, "Boot_centrality FINAL- PDirec off.RDS")

# plot centrality difference

pdf("figure 4a strength centrality difference- PDirec off.pdf", width = 5, height = 5 * 2/3)

plot(Boot_centrality, statistics = c("strength"), order = 'sample', plot = "difference", theme_bw=TRUE)

dev.off()

pdf("figure 4b closeness centrality difference- PDirec off.pdf", width = 6, height = 5 * 2/3)

plot(Boot_centrality, statistics = c("closeness"), order = 'sample', plot = "difference", theme_bw=TRUE)

dev.off()

pdf("figure 4c betweenness centrality difference- PDirec off.pdf", width = 5, height = 5 * 2/3)

plot(Boot_centrality, statistics = c("betweenness"), order = 'sample', plot = "difference", theme_bw=TRUE)

dev.off()

## case drop bootstrap

set.seed(123)

Boot_drop <- bootnet(glasso, nBoots = 5000, default = 'ggmModSelect', nCores = 8, type = "case",

statistics = c("strength", "closeness", "betweenness"))

saveRDS(Boot_drop, "Boot_case_drop FINAL- PDirec off.RDS")

# coefficient of stability measures

cor_stab <- corStability(Boot_drop)

# plot the case drop

pdf("Figure 5 net1_boot_casedrop- PDirec off.pdf", width = 5, height = 6 * 2/3)

plot(Boot_drop, statistics = c("strength", "closeness", "betweenness"))

dev.off()

####BAYESIAN INFERENCE WITH DIRECTED ACYCLIC GRAPHS####

##read data##

imputedset1 <- read.csv("Imputed 1.csv")

imputedset2 <- read.csv("Imputed 2.csv")

imputedset3 <- read.csv("Imputed 3.csv")

imputedset4 <- read.csv("Imputed 4.csv")

imputedset5 <- read.csv("Imputed 5.csv")

#get total scores

dat1 <- imputedset1[,c("CEFSA_total" ,"CAD_total", "RTD_total", "GPTS_total", "SPEQ.H_total", "GSE_total", "PTQ_total", "AIS_total", "Alexi_total", "WEMWBS_total", "SCI_total")]

dat2 <- imputedset2[,c("CEFSA_total" ,"CAD_total", "RTD_total", "GPTS_total", "SPEQ.H_total", "GSE_total", "PTQ_total", "AIS_total", "Alexi_total", "WEMWBS_total" ,"SCI_total")]

dat3 <- imputedset3[,c("CEFSA_total" ,"CAD_total", "RTD_total", "GPTS_total", "SPEQ.H_total", "GSE_total", "PTQ_total", "AIS_total", "Alexi_total", "WEMWBS_total", "SCI_total")]

dat4 <- imputedset4[,c("CEFSA_total", "CAD_total", "RTD_total", "GPTS_total", "SPEQ.H_total", "GSE_total", "PTQ_total", "AIS_total", "Alexi_total", "WEMWBS_total", "SCI_total")]

dat5 <- imputedset5[,c("CEFSA_total", "CAD_total", "RTD_total", "GPTS_total", "SPEQ.H_total", "GSE_total", "PTQ_total", "AIS_total", "Alexi_total", "WEMWBS_total", "SCI_total")]

#dat1

names(dat1)[1] <- "Dissociation"

names(dat1)[2] <- "Cognitive Appraisals"

names(dat1)[3] <- "Responses to Dissociation"

names(dat1)[4] <- "Paranoia"

names(dat1)[5] <- "Hallucinations"

names(dat1)[6] <- "General Self-Efficacy"

names(dat1)[7] <- "Perseverative Thinking"

names(dat1)[8] <- "Affect Intolerance"

names(dat1)[9] <- "Alexithymia"

names(dat1)[10] <- "Wellbeing"

names(dat1)[11] <- "Sleep"

#dat2

names(dat2)[1] <- "Dissociation"

names(dat2)[2] <- "Cognitive Appraisals"

names(dat2)[3] <- " Responses to Dissociation"

names(dat2)[4] <- "Paranoia"

names(dat2)[5] <- "Hallucinations"

names(dat2)[6] <- "General Self-Efficacy"

names(dat2)[7] <- "Perseverative Thinking"

names(dat2)[8] <- "Affect Intolerance"

names(dat2)[9] <- "Alexithymia"

names(dat2)[10] <- "Wellbeing"

names(dat2)[11] <- "Sleep"

#dat3

names(dat3)[1] <- "Dissociation"

names(dat3)[2] <- "Cognitive Appraisals"

names(dat3)[3] <- " Responses to Dissociation"

names(dat3)[4] <- "Paranoia"

names(dat3)[5] <- "Hallucinations"

names(dat3)[6] <- "General Self-Efficacy"

names(dat3)[7] <- "Perseverative Thinking"

names(dat3)[8] <- "Affect Intolerance"

names(dat3)[9] <- "Alexithymia"

names(dat3)[10] <- "Wellbeing"

names(dat3)[11] <- "Sleep"

#dat4

names(dat4)[1] <- "Dissociation"

names(dat4)[2] <- "Cognitive Appraisals"

names(dat4)[3] <- " Responses to Dissociation"

names(dat4)[4] <- "Paranoia"

names(dat4)[5] <- "Hallucinations"

names(dat4)[6] <- "General Self-Efficacy"

names(dat4)[7] <- "Perseverative Thinking"

names(dat4)[8] <- "Affect Intolerance"

names(dat4)[9] <- "Alexithymia"

names(dat4)[10] <- "Wellbeing"

names(dat4)[11] <- "Sleep"

#dat5

names(dat5)[1] <- "Dissociation"

names(dat5)[2] <- "Cognitive Appraisals"

names(dat5)[3] <- " Responses to Dissociation"

names(dat5)[4] <- "Paranoia"

names(dat5)[5] <- "Hallucinations"

names(dat5)[6] <- "General Self-Efficacy"

names(dat5)[7] <- "Perseverative Thinking"

names(dat5)[8] <- "Affect Intolerance"

names(dat5)[9] <- "Alexithymia"

names(dat5)[10] <- "Wellbeing"

names(dat5)[11] <- "Sleep"

#gaussianize

dag1<- DAGtools::gaussianize(dat1)

dag2<- DAGtools::gaussianize(dat2)

dag3<- DAGtools::gaussianize(dat3)

dag4<- DAGtools::gaussianize(dat4)

dag5<- DAGtools::gaussianize(dat5)

##run MCMC

out <- DAGtools::fit_multiple(list(dag1, dag2, dag3, dag4, dag5), scoretype = "bge", iterations=1e7)

saveRDS(out, "out FINAL.RDS")

#causal pathways for Table

sink(file = "output of DAGs FINAL.out")

DAGtools::sum_causal_paths(out,1,2, digits = 4) # 1 is dissociation

DAGtools::sum_causal_paths(out,1,3, digits = 4)

DAGtools::sum_causal_paths(out,1,4, digits = 4)

DAGtools::sum_causal_paths(out,1,5, digits = 4)

DAGtools::sum_causal_paths(out,1,6, digits = 4)

DAGtools::sum_causal_paths(out,1,7, digits = 4)

DAGtools::sum_causal_paths(out,1,8, digits = 4)

DAGtools::sum_causal_paths(out,1,9, digits = 4)

DAGtools::sum_causal_paths(out,1,10, digits = 4)

DAGtools::sum_causal_paths(out,1,11, digits = 4)

sink()

##Plotting graphs

A <- out$adj

#coefficients for nodes

n_pos <- {matrix(c(2.3,2.9, #{Dissociation};

0,4, #{Cog Apps};

0,1.3, #{Safety Behaviours}};

0,-0.9, #{Paranoia};

-3.2,0.8, #{Hallucinations};

-3,-1.3, # {GSE};

-2.3,2.8, #Perseverative thinking};

1.5,-2.6 , #Affect Intol};

3,-1.3, # {Alexithymia};

3,0.8, #Wellbeing};

-1.5,-2.6), ncol=2, byrow = TRUE)} #sleep

## coefficients for edges

bend <- matrix(0, nrow(n_pos), nrow(n_pos))

bend[1,9] = -15 #dissoc alexi

bend[1,5] = -5 #dissoc hallus

bend[2,4] = 30 #cog app para

bend[6,10] = 10 #GSE well

bend[6,7] = -10 #GSE PT

bend[7,8] = -28 #PT AI

bend[7,11] = -5 #PT sleep

bend[7,10] = 10 #PT well

bend[10,11] = 10 #well sleep

getTikzCode(A, file="plot_code_ud.tex", n_pos = n_pos, bend = bend, ud_thresh = 0.9, cutoff=0.5)

#This file (“plot_code_DAGs.tex”) then gets imported into a TeXworks script to build the final Figure
